# The Jaynes–Cummings model of a two-level atom in a single-mode para-Bose cavity field

**DOI:** 10.1038/s41598-021-02150-0

**Published:** 2021-11-24

**Authors:** H. Fakhri, M. Sayyah-Fard

**Affiliations:** Department of Theoretical Physics and Astrophysics, Faculty of Physics, University of Tabriz, P. O. Box 51666-16471, Tabriz, Iran

**Keywords:** Quantum optics, Quantum mechanics, Theoretical physics

## Abstract

The coherent states in the parity deformed analog of standard boson Glauber coherent states are generated, which admit a resolution of unity with a positive measure. The quantum-mechanical nature of the light field of these para-Bose states is studied, and it is found that para-Bose order plays an important role in the nonclassical behaviors including photon antibunching, sub-Poissonian statistics, signal-to-quantum noise ratio, quadrature squeezing effect, and multi-peaked number distribution. Furthermore, we consider the Jaynes-Cummings model of a two-level atom in a para-Bose cavity field with the initial states of the excited and Glauber coherent ones when the atom makes one-photon transitions, and obtain exact energy spectrum and eigenstates of the deformed model. Nonclassical properties of the time-evolved para-Bose atom-field states are exhibited through evaluating the fidelity, evolution of atomic inversion, level damping, and von Neumann entropy. It is shown that the evolution time and the para-Bose order control these properties.

## Introduction

Since Schrödinger introduced the most classical of single-mode quantum states so-called coherent states (CSs) as states that minimize the uncertainty relation^1^, they have played the important role in many branches of theoretical and mathematical physics, pure mathematics, and especially in quantum optics and radiophysics^2,3^. These coherent states are related to the Heisenberg-Weyl group, which later applied and generalized successfully to some models based on their Lie algebra symmetries by several authors^4–6^. In another approach discovered by Glauber^2^ for the HeisenbergWeyl group, and later generalized to some quantum models with another Lie group symmetries by Klauder^7–9^, Perelomov^10–12^, Gilmore^13,14^ and Rasetti^15^, these states reconstruct by the action of unitary displacement operator on a reference state of a group representation Hilbert space. Schrödinger’s CSs are also eigenstates of the annihilation operator of the simple harmonic oscillator algebra^2^ which in work^16^ was generalized to the eigenstates of the lowering generators of some other Lie groups. In the classical phase space, CSs as quantum analogues of points, are very close to the classical states but are still quantum in nature. Using the quasi-probabilities developed by Wigner and others, CSs allowed one to describe the behavior of light in phase space. As found by Hudson in^17^, the Wigner function for harmonic oscillator CSs is nonnegative. The state whose Wigner function has negative values in some areas of phase space is nonclassical. For any state of light the density matrix $\rho$ may be expressed in terms of a CS $|\alpha \rangle$ as $\rho =\int P(\alpha ) |\alpha \rangle \langle \alpha | d^2\alpha$, where $P(\alpha )$ is called the P-representation of the density matrix and represents a probability density function for a classical state of light, therefore, takes nonnegative values which is true for CSs. However, this function for quantum mechanical states has negative values or is a multi-peaked distribution function, such that it can no longer be interpreted as a function of probability density. There are other features that may legitimately reflect the degree of non-classicality of a given quantum state such as sub-Poissonian statistics, antibunching, and squeezing. In recent decades, several authors have proposed some extension and deformation of the bosonic Fock-Heisenberg algebra to improve some properties of quantum field theory, which most of them have been accomplished until now. A lot of researchers have introduced the various types of *q*-deformations of the simple harmonic oscillator by using Jackson’s *q*-calculus^18–21^ and considered their novel states related to *q*-deformed Lie algebras such as *q*-coherent states, *q*-squeezed states and *q*-cat states^22–29^. Another interesting modification of the Heisenberg algebra is Wigner algebra involving the reflection operator *R* and Wigner parameter $\lambda$ appeared in the bosonic commutation relations and the equations of motion. This algebra has both infinite-dimensional representations of parabosons, and finite-dimensional representations related to parafermions^30^. Concepts involving parafields and parastatistics naturally result from such generalizations^31–33^. When $\lambda$ is related to the Calogero coupling constant, this algebra is algebraic symmetry of the reduced part of the two-particle Calogero-Sutherland model or pseudo-harmonic oscillator, used for solving the quantum mechanical Calogero model^34–36^. The various systems in the field of the quantum optic can be characterized by this model^10,16,37–42^ and its *su*(1, 1) dynamical symmetry has been investigated in^11,12^. Also, when $\lambda =\frac{p-1}{2}$ this algebra is converted to the para-Bose oscillator algebra in order *p*^43–45^, which provides an appropriate formulation of particles that are neither bosons nor fermions. Moreover, for $\lambda \rightarrow 0$ it obviously reduces to an ordinary boson algebra.

On the other hand, the interaction of a quantized field with a two-level atom is a challenging problem in atom-radiation field studies which was described by the Jaynes-Cummings Hamiltonian in the electric dipole and rotating-wave approximations. This Hamiltonian is a fundamental one in quantum optics; it has an important role in the quantum description of any optical system containing the interaction between light and matter. The quantum effects in such systems with an optical field inside a cavity have been extensively studied both analytically and experimentally by many authors over the past decades (see, e.g.,^46–50^ and references therein). The Jaynes-Cummings model (JCM), which is an exactly solvable model, was first used to consider the classical effects of spontaneous emission and to follow traces of Rabi oscillations of the atomic population inversion^51^. Next, it was turned out that the Rabi oscillations collapse and revival repeatedly in a complicated pattern when the initial conditions are chosen appropriately^52^. A particular and interesting example where the collapse can be studied in detail is the case when a CS of the quantized field is applied. One main reason for the high interest in studying the dynamics predicted by JCM is the fact that the interaction of the light wave with an atom can be realized and verified experimentally in the cavity-QED setups, optical lattices, laser-cooled trapped ions and so on^53^. During the last decades, some effort has been devoted to studying possible extensions and generalizations of the standard JCM. Many authors have found that the ordinary creation and annihilation operators in original-JCM may be replaced by *q*-, *f*- and $\lambda$-deformed partners in the *q*-deformed, *f*-deformed and the parity $\lambda$-deformed one-photon JCMs, respectively^39,54^. Furthermore, Jaynes-Cummings models with intensity-dependent coupling interacting with HolsteinPrimakoff *su*(2) and *su*(1, 1) coherent states have been analysed in^55–57^ as other extensions of the standard JCM. Moreover, its numerous other extensions have been suggested and investigated such as intensity-dependent coupling, two photons or multi-photon transitions, two or three- cavity modes for three-level atoms^58^, the Jaynes-Cummings-Hubbard model^59–62^, driven Jaynes-Cummings model^63^ and the Tavis-Cummings model^64^.

The paper is organized as follows: “Unitary representation of the Para-Bose oscillator algebra” section contains a brief review of para-Bose oscillator algebra and its unitary lowest weight (Fock) representation. In “The disentangling formula for Glauber coherent states″ section, we construct the parity deformed Glauber CSs and show that they span the overcomplete, nonorthogonal basis for the corresponding para-Bose Hilbert space and are true deformations of the standard CSs associated with the harmonic oscillator system. Several nonclassical properties of these para-Bose CSs have been explored in “Nonclassical properties” section. We demonstrate that these states follow sub-, super-, and Poissonian statistics as well as photon antibunching, photon CSs and photon bunching effects, depending on the coherence and deformation parameters. Furthermore, we consider in detail the role of noncommutativity parameter $\lambda$ on other nonclassical effects, the so-called quadrature weak and strong squeezing as well as signal-to-noise ratio for *x* component and the electric field. Detailed calculations and discussions on the para-Bose light-matter interaction through the study of the Jaynes-Cummings model of a two-level atom in a single-mode para-Bose cavity field are presented in “Interaction between a two-level atom and single-mode para-Bose field” section. Time evolution of the atom-field states with the initial conditions of the Glauber coherent states and excited state for the field and atom are obtained in “Evolution of atom-field state” section. Dynamics of the fidelity and para-Bose Fock occupation distribution as well as atomic inversion and cavity damping on the collapse and revival phenomena are studied in “Evolution of the fidelity and para-Bose number distribution function and Atomic dynamics and level damping” sections, respectively. Moreover, antibunching effect, sub-Poissonian statistics, and von Neumann entropy are investigated in “Antibunched sub-Poissonian light and entangled light-mater system” section. Finally, the results are summarized in “Conclusion” section.

## Unitary representation of the Para-Bose oscillator algebra

Consider the parity operator as *R* where it is Hermitian and involutive, i.e., ${R^\dag }=R$ and $R^2=1$. A parity-deformed version of the simple harmonic oscillator algebra, the so-called pseudo harmonic oscillator algebra, characterized by a real parameter $\lambda$ is defined by the commutation relations on the generators *I*, $a, a^{\dagger }$ and $N = {a^\dag }a+\lambda (I-R)$:

1 $$\begin{aligned} \begin{array}{l} \left[ N,a\right] =-a,\qquad [N,{a^\dag }]={a^\dag },\qquad \left[ {a,{a^\dag }} \right] =F(N+1)-F(N),\\ \left[ I,a\right] =\left[ I,a^{\dagger }\right] =\left[ I,N\right] =0, \end{array} \end{aligned}$$

where *F* is an entire function satisfying $F(0)=0$, $F(n)>0$ and is equal to $F(n)=n+2\left( \left[ \frac{n+1}{2}\right] -\left[ \frac{n}{2}\right] \right) \lambda$. The symbol [.] denotes the integer part. Furthermore, the parity operator subjects to the following relations

2 $$\begin{aligned} \left\{ {R,a} \right\} = \left\{ {R,{a^\dag }} \right\} = 0, \qquad [N,R]=0. \end{aligned}$$

Let us assume that the generators of the pseudo harmonic oscillator algebra are the linear operators on the infinite-dimensional Hilbert space ${\mathcal {H}}_{\lambda }=\text{ Lin. } \text{ Span }\left\{ \left| n\right\rangle _{\lambda }|\, n\in {{\mathbb {N}}}_0\right\}$ with the orthogonal vector bases with respect to the inner product $\,_{\lambda }\langle .|.\rangle _{\lambda }$. For this generalized oscillator algebra, the state $|0\rangle _{\lambda }$ is the ground state, i.e. $a|0\rangle _{\lambda }=0$. The Fock basis states $|n\rangle _{\lambda }$ are the common eigenstates of parity operator, $R\left| n\right\rangle _{\lambda }={(-1)^n}\left| n\right\rangle _{\lambda }$, identity operator, $I|n\rangle _{\lambda }=|n\rangle _{\lambda }$, as well as number operator, $N\left| n\right\rangle _{\lambda }=n\left| n\right\rangle _{\lambda }$ and the action of the annihilation and creation operators on these states are given by

3 $$\begin{aligned} \begin{array}{l} a|n\rangle _{\lambda }=\sqrt{F(n)}\,|n-1\rangle _{\lambda },\qquad a^{\dagger }|n\rangle _{\lambda }=\sqrt{F(n+1)}\,|n+1\rangle _{\lambda }. \end{array} \end{aligned}$$

The *x*-representation of the Fock vectors $|n\rangle _{\lambda }$ are expressed in terms of the associated Laguerre polynomials^38^ as

4 $$\begin{aligned}{}&\langle x|n\rangle _{\lambda } = (-1)^{[{\frac{n}{2}}]} x^{[{\frac{n+1}{2}}]-[{\frac{n}{2}}]}|x|^{\lambda } \sqrt{\left[ {\frac{n}{2}}\right] ! \Gamma \left( \left[ {\frac{n+1}{2}}\right] +\lambda +{\frac{1}{2}}\right) } \\&\quad \times L_{[{\frac{n}{2}}]}^{[{\frac{n+1}{2}}]-[{\frac{n}{2}}]+\lambda -{\frac{1}{2}}}(x^{2}), \qquad \lambda >- {\frac{1}{2}}. \end{aligned}$$

The Eqs. (1) and (2) are also known as the algebra of the para-Bose oscillator operators of order $1+2\lambda$.

## The disentangling formula for Glauber coherent states

According to^65^, the universal disentangling formula for Glauber CSs, i.e. $|z\rangle =e^{z{a}^{\dagger }-z^{*}{a}}|0\rangle , \quad {\mathrm {for}} \quad z\in {\mathbb {C}},$ associated to the algebra (1) is given by

5 $$\begin{aligned} |z\rangle =\sum _{n=0}^{\infty }\left\{ \sum _{j=0}^{\infty }\frac{n!}{(n+2j)!}\Delta (n+1,j)(-|z|^2)^j \right\} \frac{(z{a}^{\dagger })^n}{n!}|0\rangle , \end{aligned}$$

where $\Delta (n+1,j)$ is defined as

6 $$\begin{aligned}{}&\Delta (n+1,0)=1 \\&\Delta (n+1,j)=\sum _{k_1=1}^{n+1}F(k_1)\sum _{k_2=1}^{k_1+1}F(k_2)...\sum _{k_j=1}^{k_{j-1}+1}F(k_j). \end{aligned}$$

For para-Bose algebra through inductive reasoning we derived

7 $$\begin{aligned}{}&\Delta (2n+1,j)=\frac{(n+j)!(2n+2\lambda +2j-1)!(n+\lambda )!}{2^{j-1}\,j!\, n!(2n+2\lambda )!(n+\lambda +j-1)!}, \end{aligned}$$

8 $$\begin{aligned}{}&\Delta (2n+2,j)=\frac{(n+j)!(2n+2\lambda +2j+1)!(n+\lambda +1)!}{2^{j-1}\,j!\, n!(2n+2\lambda +2)!(n+\lambda +j)!}. \end{aligned}$$

Thus we have

9 $$\begin{aligned} |z\rangle _{\lambda }&=\sum _{n=0}^{\infty }\left\{ \sum _{j=0}^{\infty }\frac{(2n)!}{(2n+2j)!}\Delta (2n+1,j)(-|z|^2)^j \right\} \frac{(za^{\dagger })^{2n}}{(2n)!}|0\rangle _{\lambda } \\&\quad +\sum _{n=0}^{\infty }\left\{ \sum _{j=0}^{\infty }\frac{(2n+1)!}{(2n+2j+1)!}\Delta (2n+2,j)(-|z|^2)^j \right\} \frac{(za^{\dagger })^{2n+1}}{(2n+1)!}|0\rangle _{\lambda }. \end{aligned}$$

Considering $\lambda$ as a nonnegative integer number, the normalized parity deformed Glauber CSs are calculated in the form of a linear combination of basic Fock-space kets as

10 $$\begin{aligned} |z\rangle _{\lambda }=\frac{2^{2\lambda }\,{\lambda !}^{\frac{3}{2}}e^{-\frac{|z|^2}{2}}}{\sqrt{(2\lambda )!}} \sum _{n=0}^{\infty }\sqrt{\frac{([\frac{n}{2}]+\lambda )!}{[\frac{n}{2}]!(n+2\lambda )!}}\,z^n\, L_{\lambda }^{[\frac{n-1}{2}]+\frac{1}{2}}(\frac{|z|^2}{2})|n\rangle _{\lambda }, \end{aligned}$$

which is in accordance with the result found in Ref.^44^. If we use the polar representation $|z| e^{i\theta }$ for complex number *z* as well as the following integral relation

11 $$\begin{aligned} \int _{0}^{\infty }|z|^{2n+1}e^{-|z|^2}\left( L_{\lambda }^{[\frac{n-1}{2}]+\frac{1}{2}}(\frac{|z|^2}{2})\right) ^2 \,d|z| =\frac{(2\lambda )!\,[\frac{n}{2}]!\,(n+2\lambda )!}{2^{4\lambda +1}\,{\lambda !}^3([\frac{n}{2}]+\lambda )!}, \end{aligned}$$

we can show that the positive measure $d{\mu }(z)=\frac{|z|}{\pi }d|z|\,d\theta$ exists such that the CSs $|z\rangle _{\lambda }$ achieve resolution of the identity condition

12 $$\begin{aligned} \int _{\mathbb {C}} d{\mu }(z)\,|z\rangle _{\lambda }\,_{\lambda }\langle z|=\sum _{n=0}^{\infty }|n\rangle _{\lambda }\,_{\lambda }\langle n|. \end{aligned}$$

Further, the overlapping of two different normalized $\lambda$-deformed CSs calculated as

13 $$\begin{aligned} \,_{\lambda }\langle z|z'\rangle _{\lambda }=\frac{{\lambda !}^32^{4\lambda }}{(2\lambda )!} e^{-\frac{|z|^2+|z'|^2}{2}} (A_++A_-), \end{aligned}$$

where

14 $$\begin{aligned} A_{\pm }=\sum _{n=0}^{\infty }\frac{(n+\lambda )!{({\bar{z}}z')}^{2n+\frac{1}{2}\mp \frac{1}{2}}}{n!(2n+2\lambda +\frac{1}{2}\mp \frac{1}{2})!} L_{\lambda }^{n\mp \frac{1}{2}}(\frac{|z|^2}{2})L_{\lambda }^{n\mp \frac{1}{2}}(\frac{|z'|^2}{2}). \end{aligned}$$

Note that it is nonzero everywhere. So the states $|z\rangle _{\lambda }$, just as the standard CSs, are overcomplete and nonorthogonal to each other. Also, it is easy to show that these generalized CSs satisfy the following equation

15 $$\begin{aligned} a|z\rangle _{\lambda }=\left( \frac{z}{2}-\frac{z}{2}R-\frac{\partial }{\partial z} -\frac{\partial }{\partial z}R\right) |z\rangle _{\lambda }. \end{aligned}$$

**Figure 1:**
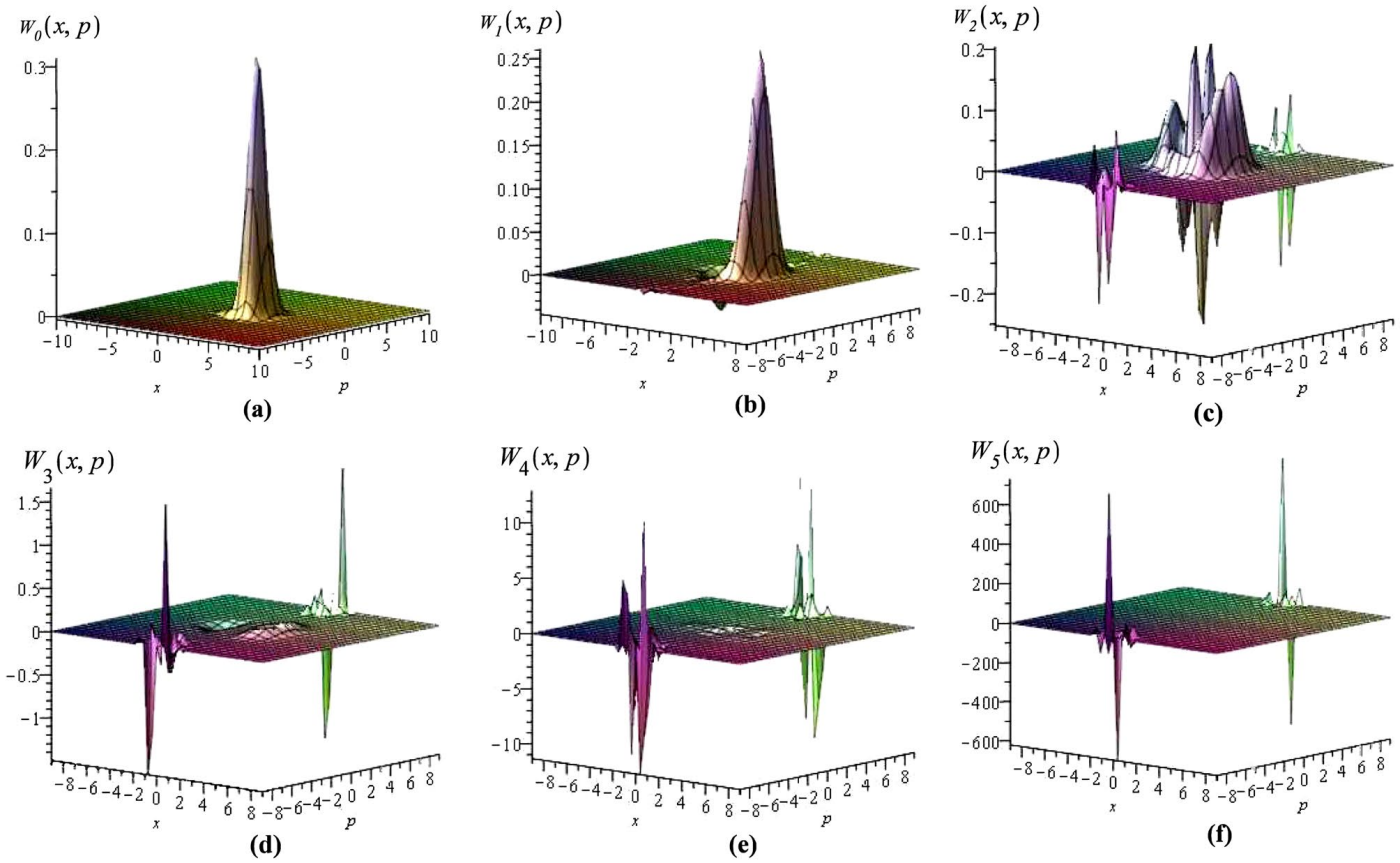
Plots of the wigner quasiprobability distribution functions $W_{\lambda }(x,p)$ corresponding to the $|z\rangle _{\lambda }$ in phase space with $z=1$ for (**a**) $\lambda =0$, (**b**) $\lambda =1$, (**c**) $\lambda =2$, (**d**) $\lambda =3$, (**e**) $\lambda =4$ and (**f**) $\lambda =5$.

Using the *x*-representation of Fock vectors (4), which is expressed in terms of the associated Laguerre polynomials, one can compute the Wigner quasiprobability distribution function for the $\lambda$-CS as

16 $$\begin{aligned} W_{\lambda }(x,p)=\frac{1}{\pi }\int _{-\infty }^{\infty }\langle x+q|{\hat{\rho }}_{\lambda }|x-q\rangle e^{-2ipq} dq, \end{aligned}$$

where ${\hat{\rho }}_{\lambda }=|z\rangle _{\lambda } \,_{\lambda }\langle z|$ is the density operator. We have plotted the Wigner function in the region $-10\le x,p\le 10$ of the phase space for $z=1$ in Fig. 1 for $\lambda =0,1,2,3,4,5$. It can be seen from the plots that the deformation remarkably changes the behavior of the phase-space distribution as it is no longer Gaussian and takes negative values which may be interpreted as a sign of nonclassical nature of the $\lambda$-CSs. By increasing $\lambda$ the height of humps of the Wigner function becomes more negative it means that the deviation of $\lambda$-CSs from classical behavior increases. Furthermore, the plots show that the Wigner function of the para-Bose oscillator is unsymmetrical under rotations of phase space.

Finally, we point out that the uncertainty relation between the generalized position and momentum operators $x=(a^{\dagger }+a)/\sqrt{2}$ and $p=i(a^{\dagger }-a)/\sqrt{2}$ with the commutation relation $[x,p]=i(1+2\lambda R)$ is not minimized by the $\lambda$-CSs. Also, the covariance between *x* and *p* via the $\lambda$-CSs is not equal to zero. Clearly, the results presented here correspond to those of the undeformed harmonic oscillator when the deformation parameter $\lambda$ tends to zero.

## Nonclassical properties

The relevant non-zero expectation values for the evaluation of nonclassical properties of the overcomplete and nonorthogonal para-Bose states $|z\rangle _{\lambda }$ are reached as

17 $$\begin{aligned}{}&\langle a\rangle _{\lambda }=z-2z\,C^{0,0,0}_{0,0,1,1}, \\&\langle a^2\rangle _{\lambda }=z^2+\frac{\lambda z^2}{|z|^2(2\lambda -1)}+\frac{z^2}{|z|^2} \,C^{0,0,0}_{1,0,1,0}, \\&\langle a^{\dagger }a\rangle _{\lambda }=|z|^2+\frac{2\lambda ^2}{2\lambda -1} +\,\lambda (2\lambda -1)\,C_{1,0,0,0}^{0,0,0}+C_{1,0,0,0}^{0,0,1}, \\&\langle {a^{\dagger }}^2 a^2\rangle _{\lambda }=|z|^4+\frac{2\lambda (2\lambda +1)}{2\lambda -1}|z|^2 +\frac{2\lambda ^2}{(2\lambda -1)(2\lambda -3)} \\&\quad + \,\lambda (\lambda -\frac{3}{2})\,C_{1,1,0,0}^{1,-2,0}+(\lambda +\frac{3}{2})C_{1,1,0,0}^{1,-2,1}, \\&\langle R\rangle _{\lambda }=-C_{0,0,0,0}^{0,0,0}, \\&\langle N\rangle _{\lambda }=|z|^2+\frac{\lambda }{2\lambda -1}+C_{1,0,1,0}^{0,0,0}, \\&\langle N^2\rangle _{\lambda }=|z|^4+\frac{6\lambda -1}{2\lambda -1}|z|^2 +\frac{2\lambda ^2}{(2\lambda -1)(2\lambda -3)} -{\lambda } (\lambda -\frac{3}{2})\,C_{1,1,0,0}^{1,-2,0} \\&\quad +(\lambda +\frac{1}{2})\,C_{1,1,0,0}^{1,-2,1}+2\,C_{1,1,2,0}^{0,0,0}, \end{aligned}$$

where

18 $$\begin{aligned} C^{m,p,q}_{i,j,k,l}=\frac{(-2)^{\lambda }\lambda !e^{-2|z|^2}}{(1+i(2\lambda -2))(1+j(2\lambda -4))} \sum _{n=0}^{\lambda }\frac{(2|z|)^{2\lambda -2n}(2j\lambda ^m+pn-1)n^q}{(-2)^n(n-k)!(2\lambda -2n+l)!}. \end{aligned}$$

The second-order intensity correlation function for zero delay time as well as the Mandel parameter are useful to characterize the antibunching and sub-Poissonian behavior of photon statistics, respectively^66,67^. These parameters for an arbitrary normalized state are defined as

19 $$\begin{aligned}{}&{\mathtt {g}}^{(2)}(0)=\frac{\langle {a^{\dagger }}^2a^2\rangle }{{\langle {a^{\dagger }}a\rangle }^2}, \end{aligned}$$

20 $$\begin{aligned}{}&{\mathtt {Q}}=\frac{{\left\langle {{N^2}} \right\rangle - {{\left\langle N \right\rangle }^2}}}{{\left\langle N \right\rangle }}-1. \end{aligned}$$

**Figure 2:**
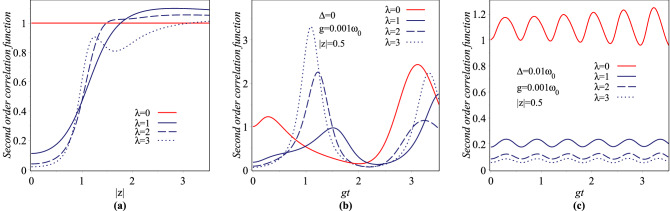
Variation of the second-order $\lambda$-correlation function for $\lambda =0, 1, 2, 3$.

**Figure 3:**
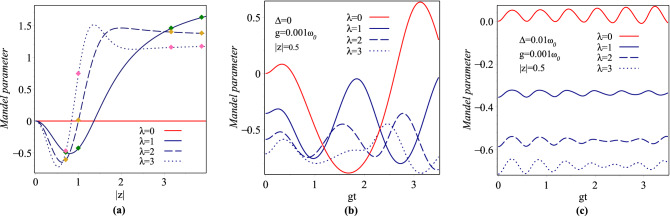
Variation of the $\lambda$-Mandel parameter for $\lambda =0, 1, 2, 3$.

Using Eqs. (17) and (18), it is straightforward to calculate the parameters $\mathtt {g}^{(2)}_{\lambda }(0)$ and $\mathtt {Q}_{\lambda }$ for the states $|z\rangle _{\lambda }$. In Figs. 2a and 3a we plot, respectively, the second order intensity $\lambda$-correlation function, $\mathtt {g}_{\lambda }^{(2)}(0)$, and $\lambda$-Mandel parameter, ${\mathtt {Q}}_{\lambda }$, versus |*z*| for $\lambda =0,1,2,3$. From Fig. 2a, we can see that, the para-Bose states $|z\rangle _{\lambda }$ demonstrate photon antibunching, photon CS and photon bunching effects in some ranges of |*z*| for a fixed value of $\lambda$. For example, for $\lambda =3$, in the ranges $0<|z|<3$, these states exhibit the antibunching effect. Also, it can be seen from Fig. 3a that all the states $|z\rangle _{\lambda }$ with the deviation from ${\mathtt {Q}}=0$, which characterizes the conventional CS, are sub-Poissonian, super-Poissonian and Poissonian in nature depending on the values of the parameters $\lambda$ and |*z*|. In^44^, Mandel $\mathtt {Q}$ parameter for some members of overcomplete, nonorthogonal para-Bose basis versus $p=1+2\lambda$ with complex parameter $|z|=\sqrt{0.5},1,\sqrt{10},\sqrt{15}$ has been drawn. The reader can check that for these values of |*z*| and for $\lambda =0,1,2,3\, (p=1,3,5,7)$, our results for the Mandel parameter, which are shown in Fig. 3a with colored symbols, are completely consistent with the ones in^44^.

**Figure 4:**
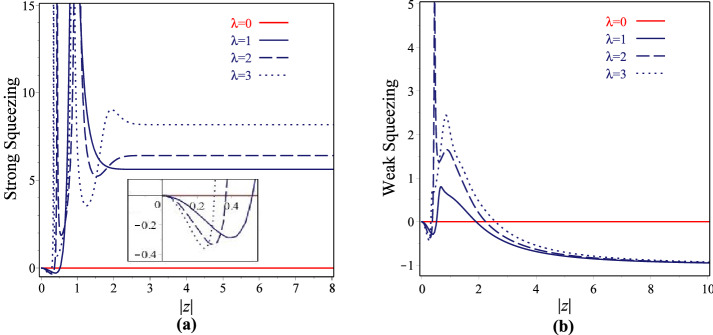
Variation of the strong and weak squeezing parameters for $\lambda$-coherent states vs |*z*| with $\theta =60$ and $\lambda =0, 1, 2, 3$.

Due to the interest of the quadrature squeezing properties, we investigate these characteristics for our $\lambda$-CSs. To this end we follow two approaches based on the Heisenberg and the Schrödinger-Robertson uncertainty inequalities lead to strong and weak squeezing, respectively^28,55,68^, in which the squeezing conditions for the quadrature *x* (*p*) are reached since the following inequalities are established

21 $$\begin{aligned}{}&S_{x(p)}^s=\frac{\sigma _{xx(pp)}-\frac{1}{2}|\langle [x,p]\rangle |}{\frac{1}{2}|\langle [x,p]\rangle |}<0, \end{aligned}$$

22 $$\begin{aligned}{}&S_{x(p)}^w=\frac{\sigma _{xx(pp)}- \sqrt{(\sigma _{xp})^2+\frac{1}{4}|\langle [x,p]\rangle |^2}}{\sqrt{(\sigma _{xp})^2+\frac{1}{4}|\langle [x,p]\rangle |^2}}<0, \end{aligned}$$

and the strongest and weakest squeezing effects are obtained when these quantities are equal to $-1$. Using the relations (17) and (18), one can obtain the strong and weak squeezing conditions for the quadrature *x* (*p*) on the $\lambda$-CSs. Figure 4a,b have been devoted to consider respectively the strong and weak squeezing parameters in *x* component associated with $\lambda$-CSs agents |*z*| for $\theta =60$ and $\lambda =0,1,2,3$. It is observed that as a consequence of the small deformations in the parameter $\lambda$ the squeezing property of *x* has been essentially changed. Although the strong squeezing for $\lambda$-CSs occurs in the small region of |*z*| but, the weak squeezing for these states can be seen in the large intervals of |*z*|. Also, the weakest squeezing effect can also be readily visualized for them.

Another nonclassical property that we examine in this section is the signal-to-quantum noise ratio which the *x*-component of it in an arbitrary normalized state is defined by

23 $$\begin{aligned} \sigma ^{(x)}=\frac{{\langle x\rangle }^2}{{\langle x^2\rangle }-{\langle x\rangle }^2}. \end{aligned}$$

**Figure 5:**
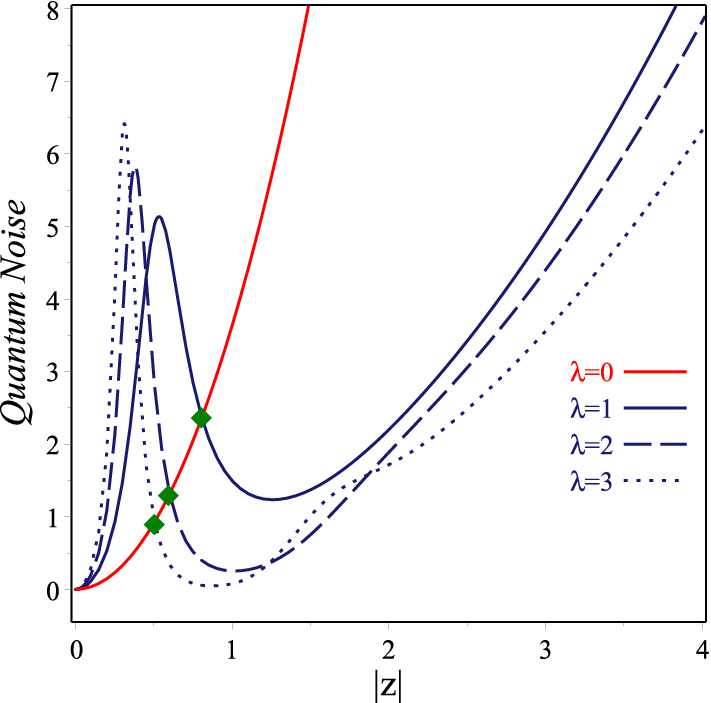
Plots of the *x*-component of signal-to-quantum noise ratios for $\lambda$-coherent states vs |*z*| with $\theta =60$ and $\lambda =0, 1, 2, 3$. The point that both undeformed and $\lambda$-deformed coherent states have the same signal-to-quantum noise ratio, are marked with the diamond symbols.

The low value of this quantity implies that the measurements in the state are noisy, whilst the high value of it indicates the clean measurements. In Fig. 5 we show the signal-to-quantum noise ratio $\sigma _{|z\rangle _{\lambda }}^{(x)}$ associated with para-Bose states $|z\rangle _{\lambda }$ versus |*z*| in the range $0<|z|<4$, for $\theta =60$ and $\lambda = 0, 1, 2, 3$. Numerical results show that for $\lambda =1,2,3$ respectively in $|z|_1\simeq 0.8,0.6,0.5$ $\lambda$-deformed CSs have the same signal-to-quantum noise ratio as undeformed ones (these points are marked with diamond symbols in figure). It is clear from the plots that in the interval $0<|z|<|z|_1$ the states $|z\rangle _{\lambda }$ are noisier than the standard CSs and vice versa in the interval $|z|_1<|z|$.

Finally, we give attention to the electric-field uncertainty. The single-photon electric field operator of the incoming wave in the ${\mathsf {z}}$-direction with the wave number *k* at each time *t* can be written in terms of quadrature operators as $E(\chi )=\frac{E_0}{\sqrt{2}}(x\cos (\chi )+p\sin (\chi ))$ where $\chi \equiv \chi ({\mathsf {z}};t)=\omega t-k{\mathsf {z}}-\frac{\pi }{2},$ and $E_0$ is the real-valued amplitude of the field. From Eqs. (17) and (18), it is easy to check that the mean field over state $|z\rangle _{\lambda }$ ($\lambda$-coherent signal) calculated as

24 $$\begin{aligned} \langle E\rangle _{\lambda }=E_0|z|\left\{ 1-2C_{0,0,1,1}^{0,0,0}\right\} \cos (\chi -\theta ), \end{aligned}$$

and the field variance, or noise, obtains as

25 $$\begin{aligned}{}&\langle (\Delta E(\chi ))^{2}\rangle _{\lambda }={\frac{E_{0}^{2}}{2}}\left\{ \left( |z|^{2}+{\frac{\lambda }{2\lambda -1}}+C_{1,0,1,0}^{0,0,0}\right) \cos (2\chi -2\theta )\right. \\&\quad -2\left( |z|-2|z|C_{0,0,1,1}^{0,0,0}\right) ^{2}\cos ^{2}(\chi -\theta ) \\&\quad \left. +|z|^{2}+{\frac{2\lambda ^{2}}{2\lambda -1}}+\lambda (2\lambda -1)C_{1,0,0,0}^{0,0,0}+ C_{1,0,0,0}^{0,0,1}-\lambda C_{0,0,0,0}^{0,0,0}+{\frac{1}{2}} \right\} . \end{aligned}$$

**Figure 6:**
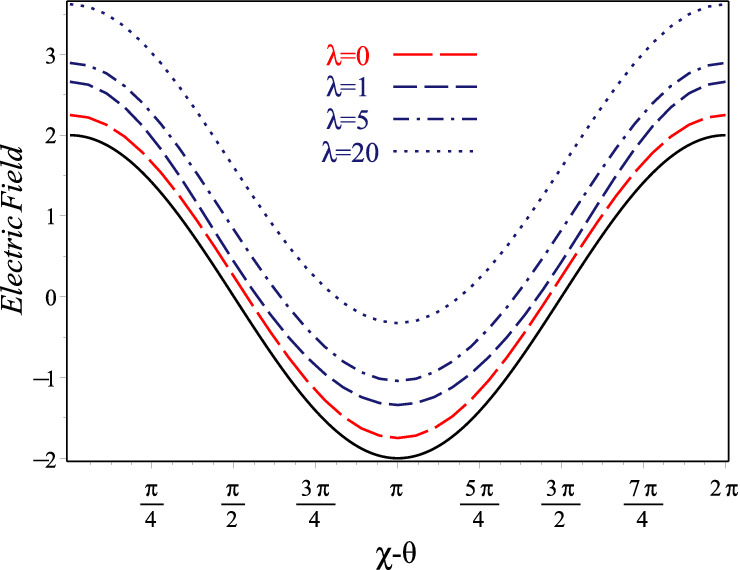
Uncertainty of electric field for $\lambda$-coherent states vs $\chi -\theta$ with $|z|=2$ and $\lambda =0, 1, 5, 20$. Solid and broken lines correspond to $\langle E(\chi )\rangle _{0}/E_0$ and $\left( \langle E(\chi )\rangle _{\lambda } +\frac{1}{2}\sqrt{\langle (\Delta E(\chi ))^2\rangle _{\lambda }}\right) /E_0$, respectively.

Here contrary to undeformed CSs the noise is phase-dependent. Now, we can get the signal-to-noise ratio as $\sigma _{|z\rangle _{\lambda }}^{(E)}=\langle E(\chi )\rangle _{\lambda }^2 /\langle (\Delta E(\chi ))^2\rangle _{\lambda }$. It is clear that for undeformed oscillator ($\lambda = 0$) with $\chi =\theta$, a maximum value of $4|z|^2$ is obtained for this quantity^69^. We have plotted the single-mode noise band $\left( \langle E(\chi )\rangle _{\lambda } +\frac{1}{2}\sqrt{\langle (\Delta E(\chi ))^2\rangle _{\lambda }}\right) /E_0$ with $|z|=2$ versus $0\le \chi -\theta <2\pi$ in Fig. 6a. This figure has been devoted to compare and consider the influence of four different values $\lambda =0, 1 ,5, 20$ on uncertainty variations of the field for state $|z\rangle _{\lambda }$. Increasing trend in the uncertainty relations with increasing $\lambda$ shown in this figure arise from the commutation relation $[E(\chi _1),E(\chi _2)]=-i{E_0^2}\sin (\chi _1-\chi _2)\,(1+2\lambda R)/2.$

## Interaction between a two-level atom and single-mode para-Bose field

Let us consider the Hamiltonian for the two-level atom coupled to the para-Bose single-mode quantized cavity field in the following form (assuming $\hbar =1$)

26 $$\begin{aligned} H_{\lambda }^{(g)}=H_0+H_{int}, \end{aligned}$$

where $H_0=\frac{\omega }{2}\{ a,{a^\dag }\}+\frac{{{\omega _0}}}{2}{\sigma _3}$ and $H_{int}=g({a}{\sigma _+}+{a^\dag }{\sigma _-})$. The first term of $H_0$ is the parity $\lambda$-deformed free-field Hamiltonian (without the zero-point energy term) that describes the energy of each photon by the parameter $\omega$ and an infinite number of the states $|n\rangle _{\lambda }$, introduced in the “Unitary representation of the Para-Bose oscillator algebra” section. The second term is the free Hamiltonian ($H_0$) corresponding to a two-level atom which the two atomic levels are separated by the energy $\omega _0$ in the Hilbert space ${\mathcal {H}}^{\mathrm {atom}}=\mathrm {Lin. \,\,Span}\{|-\rangle , |+\rangle \}$. Here, the states $|-\rangle$ and $|+\rangle$ refer to ground and excited atomic states, respectively. The three generators $\sigma _{3}=|+\rangle \langle +|-|-\rangle \langle -|$ and $\sigma _{\pm }=\sigma _1\pm i\sigma _2=|\pm \rangle \langle \mp |$ are Pauli operators associated to the atom which satisfy the *su*(2) commutation relations $[\sigma _+,\sigma _-]=\sigma _3$ and $[\sigma _3,\sigma _{\pm }]=2\sigma _{\pm }$. The Hamiltonian $H_{int}$ refers to a parity $\lambda$-deformed version of the atom-field interaction with the coupling constant *g*. The Hamiltonian (26) in the rotating-wave approximation is written as $H_{\lambda }^{(g)}=H_{JCM}^{(g)}+\frac{\omega \lambda (\lambda -R)}{2x^2}I-i\frac{g\lambda }{\sqrt{2}x}R\sigma _y$, with $I=|+\rangle \langle +|+|-\rangle \langle -|$ as identity operator on ${\mathcal {H}}^{\mathrm {atom}}$. It obviously reduces to the ordinary JCM Hamiltonian $H_{JCM}^{(g)}$ by the limiting processes $\lambda \rightarrow 0$. In the following, we will show that the fidelity between the initial state of the atom-field system and a state in every next moment is increased by increasing $\lambda$. Also, the collapse and revival phenomena in the Rabi oscillations of the atomic inversion occur with more complexity with respect to the standard JCM. Furthermore, it will be shown that by considering the decay term, the patterns of the revivals are restored as $\lambda$ is enhanced. Another effect of the deformation parameter $\lambda$ is that it reinforces the non-classical properties of the sub-Poissonian, photon antibunching and entanglement for the atom-field state $|\psi (t)\rangle _{\lambda }$.

Now, we define the orthonormal product basis for a given *n* in the Hilbert space corresponding to $\lambda$-deformed JCM Hamiltonian $H_{\lambda }^{(g)}$ as $|n,\pm \rangle \equiv |n\rangle \otimes |\pm \rangle$. Using this basis ordered as $\{|n,+\rangle ,|n+1,-\rangle \}$, one can obtain the matrix representation of $H_{\lambda }^{(g)}$

27 $$\begin{aligned} H_{\lambda ,n}^{(g)}=\left( {\begin{array}{*{20}{c}} {\omega (n+\lambda +\frac{1}{2})+\frac{{{\omega _0}}}{2}}&{}g\sqrt{F(n+1)}\\ {g\sqrt{F(n+1)}}&{}\omega (n+\lambda +\frac{3}{2})-\frac{{{\omega _0}}}{2} \end{array}} \right) \end{aligned}$$

The energy eigenvalues of $H_{\lambda ,n}^{(g)}$ are calculated as follows

28 $$\begin{aligned} E^{(g,\pm )}_{\lambda ,n}=\omega (n+\lambda +1)\pm \frac{\Omega _{\lambda ,n}^{(g)}}{2} \end{aligned}$$

where $\Omega _{\lambda ,n}^{(g)}=\sqrt{\Delta ^2+4g^2F(n+1)}$ is the generalized Rabi frequency and $\Delta =\omega _0-\omega$ is the detuning parameter. It is easy to show that the energy eigenstates are obtained as follows

29 $$\begin{aligned} \begin{array}{l} \left| {E^{(g,+)}_{\lambda ,n}} \right\rangle =\cos \theta _n{\left| {n,+} \right\rangle _{\lambda }}+\sin \theta _n{\left| {n + 1,-} \right\rangle _{\lambda }},\\ \\ \left| {E^{(g,-)}_{\lambda ,n}} \right\rangle =\sin \theta _n{\left| {n,+} \right\rangle _{\lambda }}-\cos \theta _n{\left| {n + 1,-} \right\rangle _{\lambda }}, \end{array} \end{aligned}$$

with

30 $$\begin{aligned} \sin \theta _n=\frac{{\Omega _{\lambda ,n}^{(g)}}-\Delta }{{\sqrt{{{({\Omega _{\lambda ,n}^{(g)}-\Delta })}^2}+4{g^2}F(n+1)}}}. \end{aligned}$$

**Figure 7:**
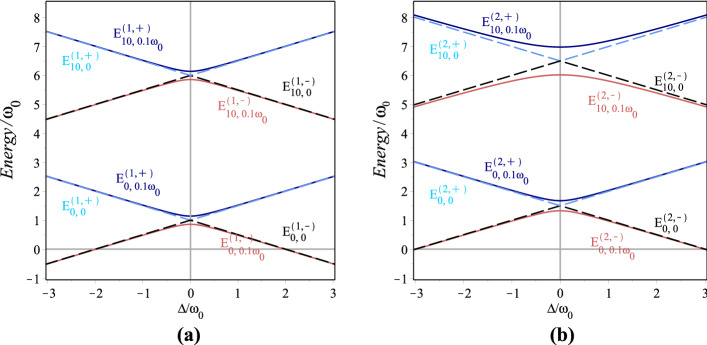
Variations of $E^{(n,\pm )}_{\lambda ,g}/\omega _0$ versus detuning parameter $-3 \omega _0<\Delta <3\omega _0$ for $\omega =0.5\omega _0$, $n=1,2$ and $\lambda =0,10$. The solid and dashed lines correspond to system with and without interaction, respectively.

It is clear that, in the limit $\lambda \rightarrow 0$, (26) reduces to the Hamiltonian of the standard JCM^70^. For the case of exact resonance, $\Delta =0$, if there is no coupling between the photon and atom ($g=0$) the eigenvalues cross each other, that is, $E^{(0,+)}_{\lambda ,n}=E^{(0,-)}_{\lambda ,n}$, whereas, for any nonvanishing value of *g*, the cross level disappears and the energy difference between the levels $E^{(0,+)}_{\lambda ,n}$ and $E^{(0,-)}_{\lambda ,n}$ is $\Omega _{\lambda ,n}^{(g)}$. The variation of the energies $E^{(g,\pm )}_{\lambda ,1}$ and $E^{(g,\pm )}_{\lambda ,2}$ as a function of the detuning parameter in the interval $-3\omega _0<\Delta <3\omega _0$ with $\omega =0.5\omega _0$ for $\lambda =0$ and 10 is represented in Fig. 7a,b, respectively. $E^{(g,-)}_{\lambda ,n}$ and $E^{(g,+)}_{\lambda ,n}$ include, respectively, lower and upper parts of the figure. In this figure, dashed lines refer to $g=0$ and full lines correspond to $g = 0.1\omega _0$. The figure shows that for even values of *n* the repulsion between energy levels of the para-Bose Jaynes-Cummings doublet increases when the deformation parameter $\lambda$ gets bigger.

### Evolution of atom-field state

To probe the dynamics of the $\lambda$-deformed JCM, we consider the so-called time-dependent interaction Hamiltonian by transforming into the interaction picture with respect to the free Hamiltonian $H_0$ as ${H_I} ={e^{i{H_0}t}}{H_{\text{int} }}{e^{ - i{H_0}t}}= g({e^{i\Delta t}}{\sigma _-}{a^{{\dag }}} + {e^{-i\Delta t}}{\sigma _+}{a}).$ We set the initial state of the $\lambda$-bosonic mode as the generalized para-Bose Glauber CSs and suppose the atom is in the excited state $|+\rangle$. So the initial state of the atomfield system is given by

31 $$\begin{aligned} {\left| {\psi (0)}\right\rangle _{\lambda }}=|z\rangle _{\lambda }\otimes |+\rangle = \sum _{n=0}^{\infty }C_{n}^{(\lambda )}(0)|n,+\rangle _{\lambda }, \end{aligned}$$

where the initial probability amplitude of the atom-field state is

32 $$\begin{aligned} C_{n}^{(\lambda )}(0)=\frac{2^{2\lambda }\,{\lambda !}^{\frac{3}{2}}e^{-\frac{|z|^2}{2}}}{\sqrt{(2\lambda )!}} \sqrt{\frac{([\frac{n}{2}]+\lambda )!}{[\frac{n}{2}]!(n+2\lambda )!}}\,z^n\, L_{\lambda }^{[\frac{n-1}{2}]+\frac{1}{2}}(\frac{|z|^2}{2}). \end{aligned}$$

Now, one can verify that the exact solution of the Schrödinger equation $i\frac{d}{dt}|\psi (t)\rangle _{\lambda }=H_I|\psi (t)\rangle _{\lambda }$ for the state vector $|\psi (t)\rangle _{\lambda }$ is

33 $$\begin{aligned} |\psi (t)\rangle _{\lambda }=\sum _{n=0}^{\infty } \left( C_{n}^{(\lambda ,+)}(t)|n,+\rangle _{\lambda }+C_{n+1}^{(\lambda ,-)}(t)|n+1,-\rangle _{\lambda }\right) , \end{aligned}$$

where

34 $$\begin{aligned} \begin{array}{l} C_{n}^{(\lambda ,+)}(t)=C_{n}^{(\lambda )}(0)\left[ {\cos (\frac{{\Omega _{\lambda ,n}^{(g)}t}}{2}) +i(\frac{\Delta }{\Omega _{\lambda ,n}^{(g)}})\sin (\frac{{\Omega _{\lambda ,n}^{(g)}t}}{2})}\right] \,{e^{-i\frac{\Delta t}{2}}},\\ \\ C_{n+1}^{(\lambda ,-)}(t)=-\frac{{2ig}}{\Omega _{\lambda ,n}^{(g)}}C_{n}^{(\lambda )}(0) \sqrt{F(n-1)+2} \,\sin (\frac{{\Omega _{\lambda ,n}^{(g)}t}}{2}) \,{e^{i\frac{\Delta t}{2}}}. \end{array} \end{aligned}$$

### Evolution of the fidelity and para-Bose number distribution function

Fidelity between the states ${\left| {\psi _1} \right\rangle }$ and ${\left| {\psi _2} \right\rangle }$ is defined as $F=|\langle \psi _2|\psi _1\rangle |^2,$ which takes the values from 0 to 1 corresponding to the minimal and maximal closeness for these states^71^. It is here considered as a transition probability from the initial state of the atom- field to another state in every next moment as

35 $$\begin{aligned} F_{\lambda }=|_{\lambda }\langle \psi (0)|\psi (t)\rangle _{\lambda }|^2= \left| \sum \limits _{n=0}^\infty {| C_n^{(\lambda )}(0){|^2}\left[ {\cos \left( \frac{{\Omega ^{(g)}_{\lambda ,n}t}}{2}\right) + i\left( \frac{\Delta }{\Omega ^{(g)}_{\lambda ,n}}\right) \sin \left( \frac{{\Omega ^{(g)}_{\lambda ,n}t}}{2}\right) } \right] }\right| ^2 \end{aligned}$$

**Figure 8:**
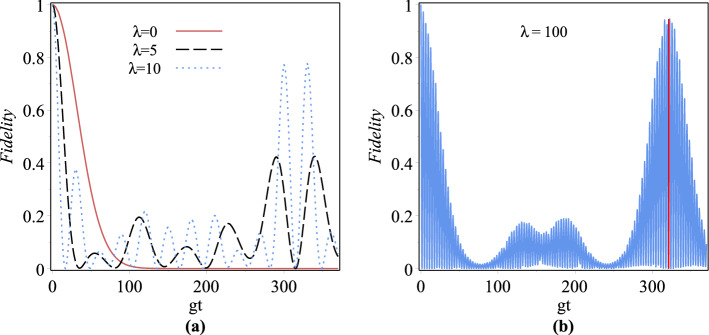
Fidelity as a function of *gt* for (**a**) $\lambda =0,5,10$ and (**b**) $\lambda =100$ with $|z|=\sqrt{5}$, $g=0.001\omega _0$ and $\Delta =0.1\omega _0$.

We plot fidelity as a function of *gt* in the interval $0<gt<370$ with $|z|=\sqrt{5}$, $\Delta =0.1\omega _0$ and $g=0.001\omega _0$ for (a) $\lambda =0,5,10$ and (b) $\lambda =100$ in Fig. 8. As can be seen in part (a) of this figure, the solid and broken lines respectively correspond to undeformed and parity deformed models. The figure indicates that with increasing $\lambda$ the fidelity increases. To obtain fidelity around 1, one needs to set the suitable parameters, for example, it obtains for $\lambda =100$ and $gt\simeq 96$ (see Fig. 8b).

**Figure 9:**
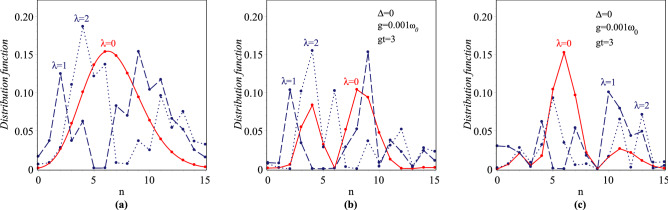
Plots of the para-Bose Fock number state distribution with $|z|=2.6$ and $\lambda =0,1,2$.

The para-Bose Fock number state distributions associated with the states $|\psi (t)\rangle _{\lambda }$ are obtained as

36 $$\begin{aligned}{}&P_{\lambda ,t}^{\pm }(n)=|\langle n,\pm |\psi _{\lambda }(t)\rangle |^2=|C_n^{(\lambda ,\pm )}(t)|^2, \end{aligned}$$

where *n* is an arbitrary non-negative integer and $P_{\lambda ,t}^{\pm }(n)$ refer to number of photons in the states $|n,\pm \rangle$ in time evolution system. One can show that these distribution functions associated with the initially state $|\psi (0)\rangle _{\lambda }$ are obtained as $P_{\lambda ,0}^{+}(n)=|C_n^{(\lambda )}(0)|^2$ and $P_{\lambda ,0}^{-}(n)=0$. Figure 9a contains the plots of changes of the $\lambda$-Boson number distribution $P_{\lambda ,0}^{+}(n)$ as a function of *n* in the interval $0\le n\le 15$ with $|z|=2.6$ for $\lambda =0,1,2$. Due to the atom and field don’t interact with each other at $t=0$, so it is obvious that these plots represent the changes in the $\lambda$-Boson number distribution function of field (or para-Bose Fock number state distribution of overcomplete and nonorthogonal states $|z\rangle _{\lambda }$). As expected, we recover the standard CSs photon distribution for $\lambda =0$ [solid red line] and more complex distributions for higher orders of $\lambda$. By increasing the deformation parameter, the plots contain significant variations with respect to the standard case and the number of peaks increases. We note that the figures of para-Bose Fock number state distribution for $\lambda =0,2\,(p=1,5)$ are in complete agreement with Fig. 1 of^44^. Also, Fig. 9b,c have been devoted to consider the $\lambda$-Boson number distribution function curve changes of time evolution system for $\Delta =0$, $g=0.001\omega _0$ and $gt=3$ associated with state $|n,+\rangle$ and $|n,-\rangle$, respectively. Both figures contain different values of deformed parameter $\lambda =0,1,2$, in which the undeformed case is denoted by the solid line and the others are shown by the broken lines. From the comparison of Fig. 9a–c, it is found that over time-leading to the decreasing (increasing) of the photon number of state $|n,+\rangle$ ($|n,-\rangle$). Furthermore, the number of peaks in plots increase, which means that the time evolution system with respect to initially one becomes more nonclassical.

### Atomic dynamics and level damping

As time goes forward, the probabilities of detecting the atom in the excited and ground states respectively are given by ${|C_{n}^{(\lambda ,+)}(t)|}^2$ and ${|C_{n+1}^{(\lambda ,-)}(t)|}^2$. To see how the atom evolves under the influence of the para-Bose CSs, it is necessary to investigate the temporal evolution of the criterion for the population inversion of collective two-level atoms. Temporal evolution of the atomic inversion for the parity $\lambda$-deformed JCM with the initial condition (31) of the atom-field state is^39^

37 $$\begin{aligned}{}&\left\langle {{\sigma _z}} \right\rangle _{\lambda }=\sum \limits _{n = 0}^\infty \left[ {|C_{n}^{(\lambda ,+)}(t)|}^2-{|C_{n+1}^{(\lambda ,-)}(t)|}^2\right] \\&\quad =\sum \limits _{n = 0}^\infty {|C_{n}^{(\lambda )}(0){|^2} \left\{ {{\left( \frac{\Delta }{\Omega _{\lambda ,n}^{(g)}}\right) ^2}+ {4F(n+1) \left( \frac{{g}}{\Omega _{\lambda ,n}^{(g)}}\right) ^2} \cos (\Omega _{\lambda ,n}^{(g)}t)} \right\} }. \end{aligned}$$

**Figure 10:**
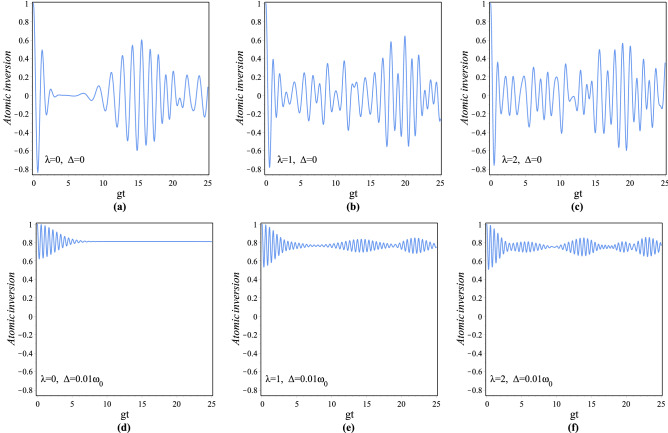
Atomic inversion $\langle \sigma _z\rangle _{\lambda }$ of the generalized JCM against the *gt* for $|z|=\sqrt{5}$, $\lambda =0, 1, 2$ and $\Delta =0, 0.01\omega _0$ with $g=0.001\omega _0$.

The quasi-periodic collapse and revival phenomena^72,73^ in atomic population inversion of the parity $\lambda$-deformed JCM against the *gt* in the interval $0<gt<25$ for $\lambda =0, 1, 2$ and $\Delta =0, 0.01\omega _0$ with $|z|=\sqrt{5}$ and $g=0.001\omega _0$ have been depicted in Fig. 10. From the figure, it is obvious that for the case of exact resonance, no clear collapse-revival phenomenon is observed for the atom-field states corresponding to nonzero deformation parameter $\lambda$. Furthermore, in the case of out-of-resonance, the partial revivals for $\lambda$-deformed field exist in a clear manner and become more periodic in comparison with the undeformed one. As we observed, by deviating from the resonance, the sequence of revivals and collapses exist and the time interval between subsequent revivals will be increased.

**Figure 11:**
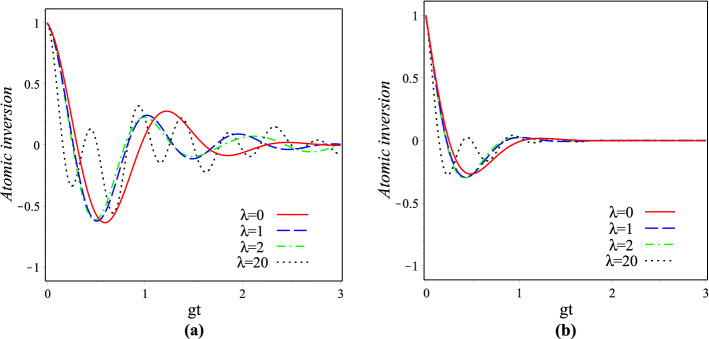
Atomic inversion $\langle \sigma _z\rangle _{\lambda }$ of the generalized JCM as a function of $0<gt<3$ with $|z|=\sqrt{5}$, $\Delta =0$ and $g=0.001\omega _0$ for (**a**) $\gamma =0.001\omega _0$ and (**b**) $\gamma =0.005\omega _0$.

The populations of excited atomic levels decay in time under several phenomena such as spontaneous emission^74^, collisions^75,76^ and scattering^77^. Regardless of the explicit dynamics of the levels populated by decay mechanisms, the finite level lifetimes can be described in good approximation by adding the appropriate non-Hermitian terms to the Hamiltonian^51,78^. Here, it is assumed that the atom is initially in the excited state, so considering the dissipation of this state, the finite level lifetimes at time *t* can be analyzed by adding phenomenological decay terms $-i\frac{\gamma }{2}|+\rangle \langle +|$ to the $H_{\lambda }^{(g)}$, where $\gamma \in {\mathbb {R}}$ is the damping constant. So by this way, using (33), the time-dependent Schrödinger equation for the atom-field state leads to

38 $$\begin{aligned}{}&{\dot{C}}_{n}^{\lambda ,+}(t)=-ig\sqrt{F(n+1)}\,e^{-i\Delta t}C_{n+1}^{(\lambda ,-)}(t)- {\frac{\gamma}{2}}{C}_{n}^{(\lambda ,+)}(t), \\&{\dot{C}}_{n+1}^{\lambda ,-}(t)=-ig\sqrt{F(n+1)}\,e^{i\Delta t}C_{n}^{(\lambda ,+)}(t), \end{aligned}$$

where dot is for derivative with respect to time. By considering the initial condition we obtain

39 $$\begin{aligned} \begin{array}{l} C_{n}^{(\lambda ,+)}(t)=C_{n}^{(\lambda )}(0)\left[ {\cos (\frac{{\Theta _{\lambda ,n}^{(g)}t}}{2}) +(\frac{2i\Delta -\gamma }{2\Theta _{\lambda ,n}^{(g)}})\sin (\frac{{\Theta _{\lambda ,n}^{(g)}t}}{2})}\right] \,{e^{-\frac{2i\Delta +\gamma }{4}t}},\\ \\ C_{n+1}^{(\lambda ,-)}(t)=-\frac{{2ig}}{\Theta _{\lambda ,n}^{(g)}}C_{n}^{(\lambda )}(0) \sqrt{F(n+1)} \,\sin (\frac{{\Theta _{\lambda ,n}^{(g)}t}}{2}) \,{e^{\frac{2i\Delta -\gamma }{4}t}}, \end{array} \end{aligned}$$

where $\Theta _{\lambda ,n}^{(g)}=\sqrt{(\Delta +i\frac{\gamma }{2})^2+4g^2F(n+1)}$. We have plotted the changes of the atomic inversion versus the scaled time in the interval $0<gt<3$ with given values $|z|=\sqrt{5}$, $g=0.001 \omega _0$ and $\Delta =0$, in Fig. 11a for $\gamma =0.001\omega _0$ and (b) for $\gamma =0.005\omega _0$ and both of them for $\lambda =0,1,2,20$. In this figure solid and broken lines respectively refer to undeformed and para-Bose models. As is seen, the Rabi oscillations of the revivals decay away in time, as the revivals will disappear sooner when $\gamma$ is increased. Furthermore, for a given $\gamma$, the patterns of revivals for the deformed case are more periodic than the undeformed case.

### Antibunched sub-Poissonian light and entangled light-mater system

The non-zero relevant expectation values for the evaluation of nonclassical properties of the cavity field over the time-evolved state Eq. (33) are

40 $$\begin{aligned} \begin{array}{lll} \langle N \rangle _{\lambda ,t}=\alpha _{\lambda }^+(t)+\alpha _{\lambda }^-(t)&{}&{} \langle N^2 \rangle _{\lambda ,t}=\beta _{\lambda }^+(t)+\beta _{\lambda }^-(t)\\ \langle a \rangle _{\lambda ,t}=\gamma _{\lambda }^+(t)+\gamma _{\lambda }^-(t)&{}&{} \langle a^2 \rangle _{\lambda ,t}=\zeta _{\lambda }^+(t)+\zeta _{\lambda }^-(t)\\ \langle a^{\dagger }a \rangle _{\lambda ,t}=\eta _{\lambda }^+(t)+\eta _{\lambda }^-(t)&{}&{} \langle {a^{\dagger }}^2a^2 \rangle _{\lambda ,t}=\xi _{\lambda }^+(t)+\xi _{\lambda }^-(t) \end{array} \end{aligned}$$

where

41 $$\begin{aligned}{}&\alpha _{\lambda }^{\pm }(t)=\sum _{n=0}^{\infty }(n+{\frac{1}{2}}\mp {\frac{1}{2}}) |C^{(\lambda ,\pm )}_{n+{\frac{1}{2}}\mp {\frac{1}{2}}}(t)|^{2} \\&\beta _{\lambda }^{\pm }(t)=\sum _{n=0}^{\infty}(n+{\frac{1}{2}}\mp {\frac{1}{2}})^{2} |C^{(\lambda ,\pm )}_{n+{\frac{1}{2}}\mp {\frac{1}{2}}}(t)|^{2} \\&\gamma _{\lambda }^{\pm }(t)=\sum _{n=0}^{\infty }C^{(\lambda ,\pm )}_{n+{\frac{3}{2}}\mp {\frac{1}{2}}}(t) {C}^{*(\lambda ,\pm )}_{n+{\frac{1}{2}}\mp {\frac{1}{2}}}(t)\sqrt{n\pm 2\lambda \left( \left[ {\frac{n}{2}}\right] -\left[ {\frac{n\mp 1}{2}}\right] \right) +{\frac{3}{2}}\mp {\frac{1}{2}}} \\&\zeta _{\lambda }^{\pm }(t)=\sum _{n=0}^{\infty }C^{(\lambda ,\pm )}_{n+{\frac{5}{2}}\mp {\frac{1}{2}}}(t) C^{*(\lambda ,\pm )}_{\pm ,n+{\frac{1}{2}}\mp {\frac{1}{2}}}(t) \sqrt{\left( F(n)+2\right) \left( F(n+1)+1\mp 1\right) } \\&\eta _{\lambda }^{\pm }(t)=\sum _{n=0}^{\infty }|C^{(\lambda ,\pm )}_{n+{\frac{1}{2}}\mp {\frac{1}{2}}}(t)|^{2}\left( n\mp 2\lambda \left( \left[ {\frac{n}{2}}\right] - \left[ {\frac{n\pm 1}{2}}\right] \right) +{\frac{1}{2}}\mp {\frac{1}{2}}\right) \\&\xi _{\lambda }^{\pm }(t)=\sum _{n=0}^{\infty }|C^{(\lambda ,\pm )}_{n+{\frac{1}{2}}\mp {\frac{1}{2}}}(t)|^{2} F(n)\left( F(n-1)+1\mp 1\right) . \end{aligned}$$

Now, one can immediately evaluate the parameters $\mathtt {g}^{(2)}_{\lambda ,t}(0)$ and $\mathtt {Q}_{\lambda ,t}$ for the normalized time-evolved atom-field state $|\psi (t)\rangle _{\lambda }$ as

42 $$\begin{aligned}{}&\mathtt {g}^{(2)}_{\lambda ,t}(0)=\frac{\xi _+^{\lambda }(t)+\xi _-^{\lambda }(t)}{\left( \eta _+^{\lambda }(t)+\eta _-^{\lambda }(t)\right) ^2} \\&\mathtt {Q}_{\lambda ,t}=\frac{\beta _{\lambda }^+(t)+\beta _{\lambda }^-(t)-\left( \alpha _{\lambda }^+(t)+\alpha _{\lambda }^-(t)\right) ^2}{\alpha _{\lambda }^+(t)+\alpha _{\lambda }^-(t)}-1 \end{aligned}$$

Figure 2b,c show the plots of function $\mathtt {g}_{\lambda ,t}^{(2)}(0)$ versus *gt* for $\Delta =0$ and $0.01\omega _0$, respectively, with $|z|=0.5$, $g=0.001\omega _0$ and $\lambda =0,1,2,3$. It is obvious from figures that, for the case of exact resonance, the states $|\psi (t)\rangle _{\lambda }$ demonstrate photon antibunching, photon CSs, and photon bunching effects in some ranges of time for both deformed and undeformed cases of the field. But, in the case of out-of-resonance, these states exhibit just photon antibunching effect when $\lambda$ increases. Also, we have plotted the Mandel parameters $\mathtt {Q}_{\lambda ,t}$ in terms of *gt* for time evolution system with $\Delta =0$ and $0.01\omega _0$ in Fig. 3b,c, respectively. Each of the parts (b) and (c) involves four different curves corresponding to the deformation parameters ${\lambda =0,1,2,3}$ with $|z|=0.5$ and $g=0.001\omega _0$. For both resonant and out-of-resonant cases, there are the sub-, supper- and Poissonian statistics for the undeformed oscillator, whereas, when the deformation parameter $\lambda$ is increased from 0 to 3, the sub-Poissonian statistic as a nonclassical behavior of the atom-field state $|\psi (t)\rangle _{\lambda }$ becomes stronger and supper-Poissonian as well as Poissonian statistics of it disappear.

**Figure 12:**
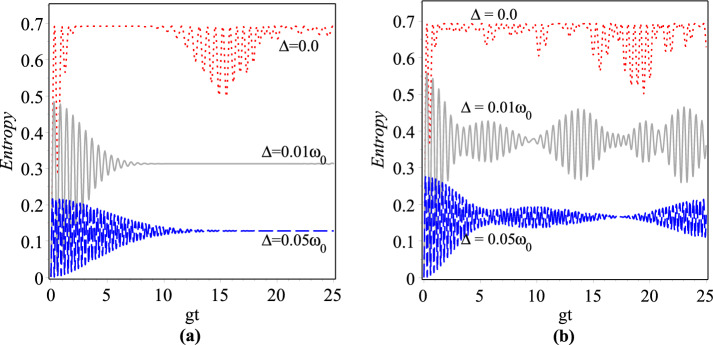
Plots of the entropy versus gt with $g=0.001\omega _0$ and $|z|=\sqrt{5}$ for (**a**) $\lambda =0$, (**b**) $\lambda =2$.

Quantum entanglement is one of the striking features which plays a crucial role in the development of quantum information theory^79^ such as quantum computations^80,81^, cryptography^82^ and quantum teleportation^83^. Here we pay attention to the time evolution of entanglement, which can be quantified by the von Neumann entropy. The von Neumann entropy for given density operator $\rho$ is defined as^84^:

43 $$\begin{aligned} S(\rho )=-Tr({\rho }\ln {\rho }), \end{aligned}$$

in which, the Boltzmann constant is assumed to be unity. This quantity is equal to 0 and 1 for all pure and maximally entangled states, respectively, whereas for a statistical mixture the entropy is $0<S<1$. Here, we assume that the function $\rho _{_{AF}}(t)$ is the density operator for the atom-field system and the partial trace of it over ${\mathcal {H}}^{\mathrm {atom}}$ (${\mathcal {H}}_{\lambda }$) gives the reduced density matrix of the atom (field): $\rho _{_{A}}(t)={\mathrm {tr}}_{_{F}}\rho _{_{AF}}(t)$ $\left( \rho _{_{F}}(t)={\mathrm {tr}}_{_{A}}\rho _{_{AF}}(t)\right)$. The dimensionless partial entropy of von Neumann for the atom (field) in terms of its corresponding density matrix is given by

44 $$\begin{aligned} S_{_{A}(F)}(t)=-{\mathrm {tr}}_{_{A}(F)}(\rho _{_{A}(F)}(t)\ln \rho _{_{A}(F)}(t)). \end{aligned}$$

The density matrix for the pure state (33) is $\rho _{_{AF}}(t)=\left| \psi (t)\rangle _{\lambda }\,_{\lambda } \langle \psi (t)\right|$. The real positive Schmidt weights $\lambda _+=\sum \nolimits _{n = 0}^\infty \left| C_{n }^{(\lambda ,+)}(t)\right| ^2$ and $\lambda _-=\sum \nolimits _{n = 0}^\infty \left| C_{n + 1 }^{(\lambda ,-)}(t)\right| ^2$ are the common eigenvalues of the reduced density matrices $\rho _{_{A}}(t)$ and $\rho _{_{F}}(t)$ so that they obey $\lambda _++\lambda _-=1$ which, in turn, shows that the Schmidt rank of the bipartite pure state $\left| {\psi (t)} \right\rangle _{\lambda }$ is 2 in any next time. Therefore, as time goes far away from the initial moment entanglement between the atom and field creates, which the degree of it can be characterized by the same entropy for the reduced density matrices of the atom and field:

45 $$\begin{aligned} S_{_A(F)}(t)=-\lambda _+\ln \lambda _+-\lambda _-\ln \lambda _-. \end{aligned}$$

According to the initial conditions we have $S_{_A}(0)=0$, that’s mean the system of atom-field is separable, which is also verified by Fig. 12. Figure 12a,b contain the plots of changes of the entropy against the scaled time $0<gt<25$ for $g=0.001\omega _0$, $|z|=\sqrt{5}$ and three different values of detuning parameter $\Delta =0,\,0.01\omega _0,\,0.02\omega _0$ with the deformation parameters $\lambda =0$ and $\lambda =2$, respectively. As it is seen from figures, the time evolution of atomic entropy has quasi-regular oscillatory behaviors in both resonant and off-resonant conditions as well as in both $\lambda$-deformed and undeformed cases. A comparison between Figs. 10 and 12 shows that the partial revivals and the partial entropies of the atom-field system have the same oscillation patterns and are modulated in the Rabi frequency. From the comparison of Fig. 12a with Fig. 12b in a given time interval, we conclude that increasing of $\lambda$ causes the number of peaks for the quasi-oscillations of the von Neumann entropies as well as their height to be increased. This, in turn, implies that more entanglement occurs by increasing the deformation parameter $\lambda$. The reader can easily examine that the dynamics of atom-field interaction are the same in both the above resonance (blue) and below resonance (red) detuning of the incident light. It means that the results obtained for $\Delta >0$ are the same for $\Delta <0$.

## Conclusion

The results are summarized in this section as follows: The parity deformed Glauber CSs have been created by acting $\lambda$-displacement operator onto the vacuum state of the para-Bose oscillator algebra of order $p=2\lambda +1$. We have obtained an appropriate measure in order to realize the resolution of the identity condition and have shown that the $\lambda$-CSs satisfy a $\lambda$-deformed counterpart of the eigenvalue equation of the annihilation operator. $\lambda$-dependency of the interference patterns of the parity deformed Glauber coherent states have been investigated and cleared by evaluating the Wigner quasiprobability distribution function. We have demonstrated that the deformation parameter has an important role to change the behavior of the phase-space distribution so that by increasing $\lambda$, it is no longer Gaussian and takes negative values which may be interpreted as a signature of quantumness. Besides considering the $\lambda$-deformed Glauber CSs generation, we were focused on the quantum-mechanical nature of the light field of them as the occurrence of photon antibunching, sub-Poissonian statistics, quadrature squeezing, and signal-to-quantum noise ratio as well as on theirs interactions with matter. We were found that these states obey every three types of statistics and demonstrate photon antibunching, photon CSs, and photon bunching effects depending on the value of the parameters $\lambda$ and |*z*|. Moreover, we have used two different types of squeezing degrees based on the Heisenberg and Schrödinger-Robertson uncertainty relations to measure the degree of quadrature squeezing. We have shown that the strong and weak squeezing for the quadrature *x* respectively occur in the small and large region of |*z*| and there are the weakest squeezing effect associated with para-Bose CSs. It has been confirmed through the evaluation of signal-to-quantum noise ratio that in the intervals $0<|z|<|z|_1$ and $|z|_1<|z|$ the outputs of detectors corresponding to the states $|z\rangle _{\lambda }$ in comparison with the undeformed oscillator case may be led to good and poor optical system performances, respectively. For these states, we have also obtained the variance of the electric single-mode field operator and found that the variance generally increases when $\lambda$ increases from 0 to larger values. Then, we have analyzed the quadrature component distributions and showed that there are multi-peaked number distributions as nonclassical properties.

Finally, we have considered the interaction between the light field of the para-Bose Glauber CSs and matter as a two-level atom in the framework of the JCM and obtained time-evolved atom-field states. In the case of off-resonance, it has been shown that the height of the peaks in the quasi-oscillations of the fidelity increase by increasing $\lambda$. We have showen how an appropriate choice of parameters allows one to find fidelity around one. It has investigated that the partial revivals of the Rabi oscillations in the case of resonance for $\lambda \ne 0$ become less distinct with increasing the time, whilst those for the simple harmonic oscillator with $\lambda =0$ are regular and complete. But, in out-of-resonance cases, the partial revivals become thinner and more periodic in case that $\lambda$ does not vanish. Indeed, in both resonance and out-of-resonance cases, the revival times turn longer when $\lambda$ increases. These partial revivals of the Rabi oscillations by adding a decay term to the interaction Hamiltonian disappear. Another important result which we have obtained is that the oscillation patterns for both partial revivals and the partial entropies of the atom-field system are the same and are modulated in the Rabi frequency. Furthermore, the number of the peaks for the quasi-oscillations of the von Neumann entropies as well as their height are increased by increasing $\lambda$, which, in turn, is an indication for more entanglement. Also, the other nonclassical behaviors of the time-evolved state through the evaluation of photon number distribution, second-order correlation functions and Mandel’s parameter have been confirmed and the influence of the deformation on them investigated.
